# The temporal behavior and consistency of bipolar atrial electrograms in human persistent atrial fibrillation

**DOI:** 10.1007/s11517-017-1667-1

**Published:** 2017-07-03

**Authors:** Tiago P. Almeida, Gavin S. Chu, Michael J. Bell, Xin Li, João L. Salinet, Nawshin Dastagir, Jiun H. Tuan, Peter J. Stafford, G. André Ng, Fernando S. Schlindwein

**Affiliations:** 1 Department of Engineering, University of Leicester, University Road, Leicester, England LE1 7RH UK; 2 Engineering, Modelling and Applied Social Sciences Centre, Federal ABC University, Santo André, Brazil; 3 Department of Cardiovascular Science, University of Leicester, Leicester, UK; 4 University Hospitals of Leicester NHS Trust, Leicester, UK; 5 National Institute for Health Research Leicester Cardiovascular Biomedical Research Centre, Glenfield Hospital, Leicester, UK

**Keywords:** Atrial fibrillation, Fractionation, Catheter ablation, Electrophysiology mapping, Stability

## Abstract

The unstable temporal behavior of atrial electrical activity during persistent atrial fibrillation (persAF) might influence ablation target identification, which could explain the conflicting persAF ablation outcomes in previous studies. We sought to investigate the temporal behavior and consistency of atrial electrogram (AEG) fractionation using different segment lengths. Seven hundred ninety-seven bipolar AEGs were collected with three segment lengths (2.5, 5,and 8 s) from 18 patients undergoing persAF ablation. The AEGs with 8-s duration were divided into three 2.5-s consecutive segments. AEG fractionation classification was applied off-line to all cases following the CARTO criteria; 43% of the AEGs remained fractionated for the three consecutive AEG segments, while nearly 30% were temporally unstable. AEG classification within the consecutive segments had moderate correlation (segment 1 vs 2: Spearman’s correlation *ρ* = 0.74, kappa score *κ* = 0.62; segment 1 vs 3: *ρ* = 0.726, *κ* = 0.62; segment 2 vs 3: *ρ* = 0.75, *κ* = 0.68). AEG classifications were more similar between AEGs with 5 and 8 s (*ρ* = 0.96, *κ* = 0.87) than 2.5 versus 5 s (*ρ* = 0.93, *κ* = 0.84) and 2.5 versus 8 s (*ρ* = 0.90, *κ* = 0.78). Our results show that the CARTO criteria should be revisited and consider recording duration longer than 2.5 s for consistent ablation target identification in persAF.

**Electronic supplementary material:**

The online version of this article (doi:10.1007/s11517-017-1667-1) contains supplementary material, which is available to authorized users.

## Introduction

During atrial fibrillation (AF), atrial electrograms (AEGs) with low amplitude and multiple activations are thought to represent atrial substrate, with structural and electric remodeling [7]. Complex fractionated atrial electrograms (CFAEs) have been introduced as markers of such atrial sites and, therefore, targets for ablation [7]. CFAE-guided ablation has become broadly used as an adjunctive therapy to pulmonary vein isolation (PVI) for persistent atrial fibrillation (persAF) [2]. However, the low reproducibility of outcomes [1] and recent evidence that CFAE ablation additional to PVI does not improve the ablation outcome [17] motivated intense debate on the meaning of the atrial substrate represented by CFAEs. Moreover, there is no consensus about the spatiotemporal dynamics of the underlying mechanisms of AF [12]. While some works have shown that AEG fractionation has a high degree of spatial and temporal stability [8, 11, 18], others suggested that CFAEs are temporally variable [6], which might be one of the reasons for the inconsistency in CFAE-guided ablation outcomes in persAF patients.

The two commercial systems more frequently used for automated CFAE classification are the Ensite NavX™ (St. Jude Medical, St. Paul, MN) and the CARTO (Biosense Webster, Diamond Bar, CA). In a recent work, we have investigated discordances in automated classification of CFAEs performed by those systems [1].

Previous works have investigated different segment lengths to characterize CFAEs using NavX—since this system allows for different AEG duration recordings (1 to 8 s)—suggesting AEG duration of 5 s or longer to consistently measure CFAEs [14]. Since its introduction circa 10 years ago, CARTO inherently limits the AEG collection to 2.5 s [13]. Consequently, few studies investigated segment length to assess fractionation using the CARTO criteria. To the best of our knowledge, only one study has shown that different AEG lengths might influence CFAE classification with the CARTO criteria, mostly using qualitative data [15]. In the present study, we investigated the spatiotemporal behavior of AEGs according to the CARTO criteria for CFAE classification considering consecutive AEGs with 2.5 s and also by investigating AEG fractionation using AEGs with 2.5, 5, and 8 s.

## Methods

### Study population

The population consisted of 18 patients (16 male; mean age 56.1 ± 9.3 years; history of AF 67.2 ± 45.6 months) referred to our institution for first-time catheter ablation of persAF [16]. Details of the clinical characteristics of the study subjects are provided in the Supplementary material. All patients were in AF at the start of the procedure. Study approval was obtained from the local ethics committee and all procedures were performed with full informed consent.

### Electrophysiological study

All antiarrhythmic drugs, except amiodarone, were discontinued for at least 5 half-lives before the start of the procedure. Details of the mapping procedure have been described previously [16]. Briefly, 3D left atrial (LA) geometry was created within NavX using a deflectable, variable loop circular pulmonary vein (PV) mapping catheter (Inquiry Optima, St. Jude Medical). PVI was performed with a point-by-point wide area circumferential ablation approach (Cool Path Duo irrigated RF catheter, St. Jude Medical), followed by the creation of a single roof line (RL). PVI was defined as the abolition of electrical signals on the circular mapping catheter when positioned within each PV.

No additional ablation targeting CFAE was performed in this study. CFAE mapping was performed for further off-line analyses. Sequential point-by-point bipolar AEGs were collected also using the Inquiry Optima from 15 predetermined atrial regions before and after PVI and RL creation (PVI + RL) [16]. All patients were in AF before and after PVI + RL during signal collection. Sinus rhythm was achieved either by PVI + RL or through DC cardioversion.

### Signal analysis

A total of 797 AEGs were recorded from the LA, 455 before and 342 after PVI (1200 Hz sampling frequency; 30–300 Hz band-pass filter; 50 Hz Notch filter). Some patients had more AEGs collected than others, as shown on the Table 1. Two patients (8 and 10) account for 24% of all AEGs collected. Nevertheless, each of the remaining patients accounts for a similar number of AEGs collected, which still provide a good representation of the population in this study.

**Table 1:** Number (and percentage) of AEGs collected per patient, before and after ablation

	Pre-ablation		Post-ablation		Total	
	No.	%	No.	%	No.	%
Patient 1	17	4	11	3	28	4
Patient 2	21	5	17	5	38	5
Patient 3	17	4	9	3	26	3
Patient 4	18	4	12	4	30	4
Patient 5	34	7	28	8	62	8
Patient 6	24	5	16	5	40	5
Patient 7	28	6	20	6	48	6
Patient 8	49	11	54	16	103	13
Patient 9	26	6	22	6	48	6
Patient 10	53	12	31	9	84	11
Patient 11	19	4	12	4	31	4
Patient 12	15	3	12	4	27	3
Patient 13	27	6	20	6	47	6
Patient 14	15	3	7	2	22	3
Patient 15	33	7	20	6	53	7
Patient 16	16	4	15	4	31	4
Patient 17	16	4	16	5	32	4
Patient 18	27	6	20	6	47	6
Total	455		342		797	

The LA maps of the 18 patients were segmented by an experienced clinician into six regions—the PVs (outside the PV junctions and PVI lesions), roof, posterior, anterior, septum, and lateral. The analyses described below consider the combined data before and after PVI + RL (total 797 AEGs). Detailed analyses before and after PVI + RL are provided in the Supplementary material.

### The CARTO algorithm (CARTO 3 system, 2008–2014, version 4.3)

A detailed description of the CARTO system is provided elsewhere [1]. Briefly, CARTO provides 3D representation of the atrium and online automated CFAE detection based on complex intervals between successive peaks and troughs occurring inside a fixed 2.5-s window of sequentially recorded bipolar AEGs. The number of identified complex intervals is referred to as the interval confidence level (ICL) and characterizes the repetitiveness of the CFAE complexes. CARTO software also finds, as complementary indices, the average of the identified interval, referred to as the average complex interval (ACI), and the shortest identified interval, referred to as the shortest complex interval (SCI).

Typically, ICL < 4 represents low fractionation, 4 ≤ ICL < 7 refers to moderate fractionation, and ICL ≥ 7 indicates high fractionation.

### Temporal consistency of AEG fractionation with different segment lengths

**Fig. 1:**
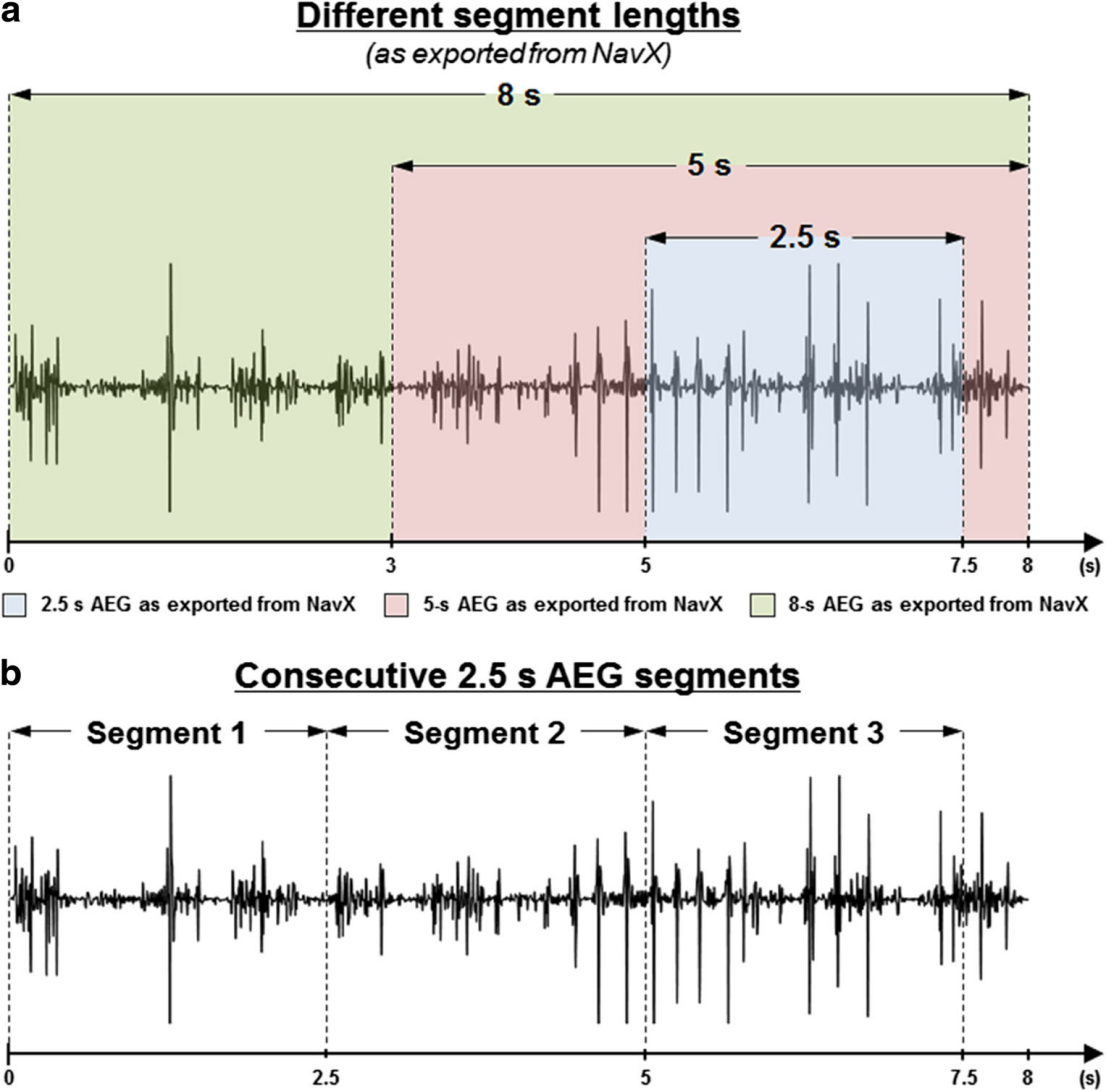
**a** Different segment lengths exported from NavX to investigate the temporal consistency of AEG fractionation. The maximum AEG length that can be exported from NavX is 8 s, and smaller segments are exported as portions of the original 8 s. **b** The 8-s segments were divided in three consecutive 2.5-s segments, as illustrated in **b**, accordingly: 0 to 2.5 s; 2.5 to 5 s; 5 to 7.5 s

CFAE classifications performed in AEGs with different segment lengths have been analyzed to investigate the temporal consistency of AEG fractionation. The configuration of the different segment lengths as exported from NavX is illustrated in Fig. 1a. The maximum AEG length that can be exported from NavX is 8 s, and shorter segments were exported as portions of the original 8 s.

To overcome the CARTO’s limitation of AEGs with fixed 2.5-s window, each AEG was exported from NavX with three segment lengths (2.5, 5, and 8 s). For each case, ICL, ACI, and SCI were calculated using a validated off-line MATLAB algorithm [1]. Since currently ICL thresholding for CFAE classification as defined by CARTO is referred to a default 2.5-s segment length (ICL ≥ 4), there is no validated ICL threshold for CFAE classification using segment lengths longer than 2.5 s. Therefore, the ICL calculated for the 5-s segment lengths was normalized by a factor of 2, while the ICL calculated for the 8-s segment lengths was normalized by 3.2. For instance, if ICL = 8 inside a 5-s segment, the normalized ICL is 4 for a corresponding 2.5-s segment. Similarly, if ICL = 12 inside an 8-s segment, the normalized ICL is also 4, approximately. That allowed a head-to-head comparison between ICL as measured with 2.5, 5, and 8 s. Bland-Altman plots were created to assess the average difference (bias) from the ICL, ACI, and SCI measured with the different segment lengths.

### Temporal behavior of consecutive AEGs

Consecutive AEG segments were assessed to infer about AEG temporal behavior. For each AEG, the 8-s segments were divided in three consecutive 2.5-s segments, as illustrated in Fig. 1b, accordingly: 0 to 2.5 s, 2.5 to 5 s, and 5 to 7.5 s. Therefore, three consecutive segments with 2.5-s length were created for each one of the 797 AEGs, allowing the investigation of the temporal behavior in the same points. The ICL, ACI, and SCI were measured for each segment also using the validated off-line MATLAB algorithm [1], and each segment was compared to the other. A best fit exponential was computed to estimate the time constant of stable AEGs according to GraphPad Prism 6’s (©2014 GraphPad Software, La Jolla, CA) One Phase Decay best fit.

### Statistical analysis

All continuous nonnormally distributed variables are expressed as median ± interquartile interval. Nonparametric paired multiple data were analyzed using the Friedman test with Dunn’s correction.

Spearman’s correlation (*ρ*) was calculated to quantify the correlation between AEG classifications measured with different segment lengths (2.5, 5, and 8 s) and the correlation between AEG classifications measured within the three consecutive segments. The agreement of CFAE classification performed by ICL—either measured with different segment lengths (2.5, 5, and 8 s) or within the three consecutive segments—was assessed by the Cohen’s kappa (*κ*) score with four ICL thresholds (ICL ≥ 4, 5, 6, 7) [5]. A kappa score within the range 0 ≤ *κ* < 0.4 suggests marginal agreement between two indices, 0.4 ≤ *κ* ≤ 0.75 good agreement, and *κ* > 0.75 excellent agreement. *P* values of less than 0.05 were considered statistically significant.

## Results

### Temporal behavior of consecutive AEGs

**Fig. 2:**
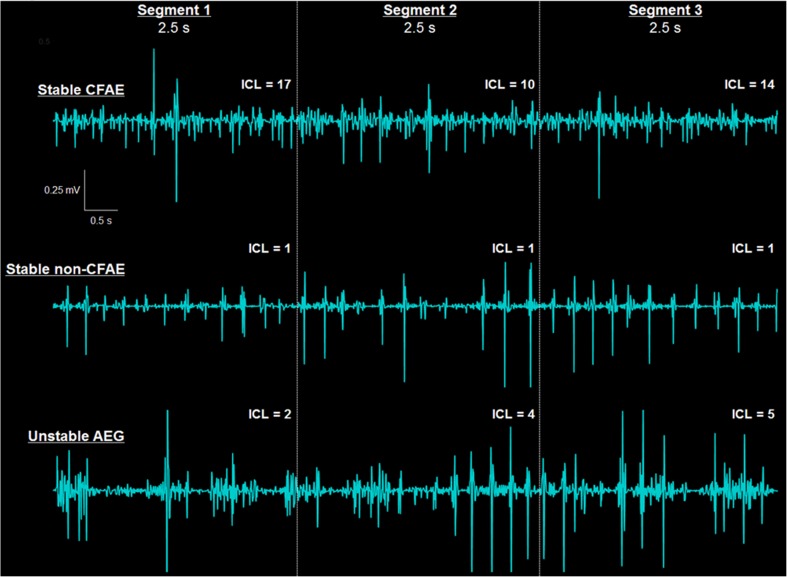
Illustration of the different types of AEGs found when analyzing the consecutive AEG segments. Stable CFAEs (*upper trace*) are AEGs with ICL ≥ 4 in all assessed segments (*upper trace*). Stable non-CFAEs (*middle trace*) are AEGs with ICL < 4 in all assessed segments. Unstable AEG (*lower trace*) are AEGs with ICL varying to/from ICL ≥ 4 to ICL < 4 within the assessed segments

**Fig. 3:**
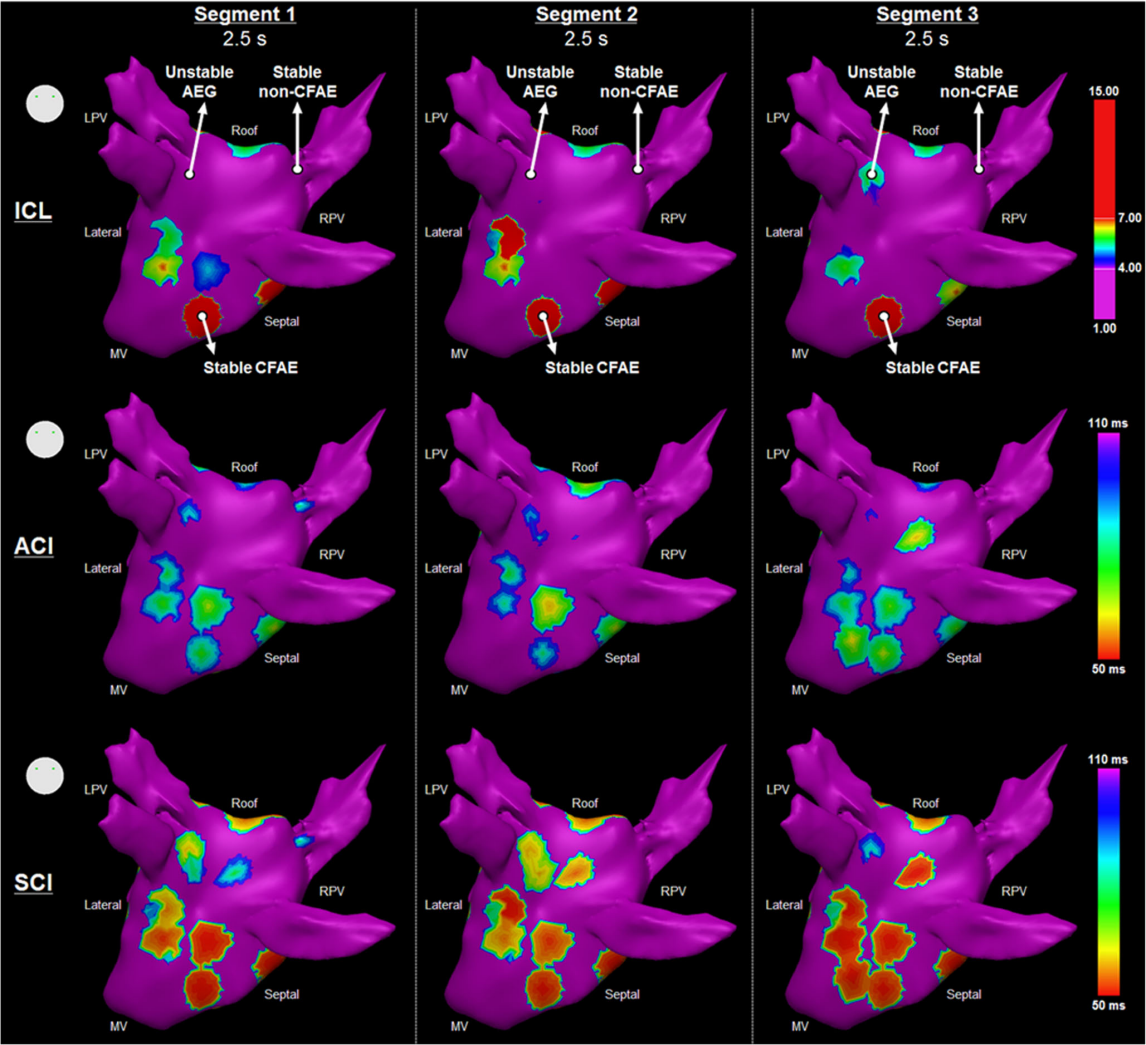
The resultant LA maps based on the three consecutive AEG segments with 2.5-s duration each for one patient as measured by the ICL (*upper*), the ACI (*middle*), and the SCI (*bottom*). The location of the AEGs classified as stable CFAE, stable non-CFAE, and unstable shown in Fig. 2 are marked in the ICL map

Three types of AEGs have been identified when investigating the consecutive segments, as illustrated in Fig. 2: “stable CFAEs” as AEGs with ICL ≥ 4 in all assessed segments, “stable non-CFAEs” as AEGs with ICL < 4 in all assessed segments, and “unstable AEG” as AEGs with ICL varying to and from ICL ≥ 4 to ICL < 4 within the assessed segments. Each AEG segment also affected the resulting CFAE map as generated by ICL, ACI, and SCI (Fig. 3). The locations of the AEGs classified as stable CFAE, stable non-CFAE, and unstable shown in Fig. 2 are also marked in the ICL map in Fig. 3.

Moderate correlation was found in the AEG classification performed by ICL, ACI, and SCI, measured in each consecutive segment, as shown in Table 2. The average correlation for ICL within the three segments was *ρ* = 0.74 ± 0.01 (mean ± SD), while ACI was *ρ* = 0.44 ± 0.02, and SCI was *ρ* = 0.55 ± 0.03. The average agreement of CFAE classification performed by ICL ≥ 4 between segments was *κ* = 0.64 ± 0.04 and deteriorated when increasing ICL threshold.

**Table 2:** Spearman’s correlation for ICL, ACI, and SCI and kappa score for CFAE classification with different thresholds measured from the consecutive 2.5-s AEG segments

2.5-s AEG segment		1 vs 2	1 vs 3	2 vs 3	*P* value
Spearman’s correlation (*ρ*)	ICL	0.735	0.726	0.748	<0.0001
	ACI	0.455	0.430	0.421	<0.0001
	SCI	0.554	0.521	0.569	<0.0001
Kappa score (*κ*)	ICL ≥ 4	0.616	0.620	0.676	<0.0001
	ICL ≥ 5	0.591	0.594	0.651	<0.0001
	ICL ≥ 6	0.505	0.507	0.555	<0.0001
	ICL ≥ 7	0.465	0.474	0.489	<0.0001

**Fig. 4:**
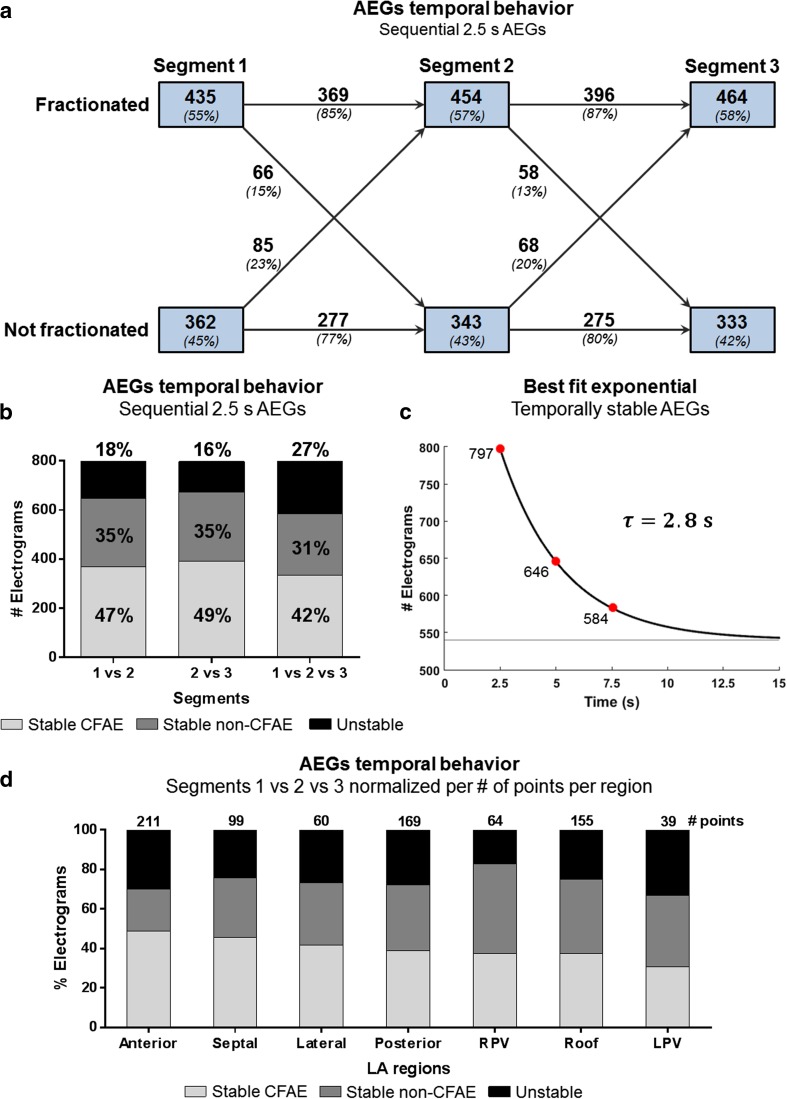
The temporal behavior of three consecutive AEGs segments with 2.5-s duration each. **a** The 8-s segments were divided in three consecutive 2.5-s length segments. The ICL was measured in each segment and classified whether as fractionated or nonfractionated. The AEGs that remained fractionated, remained nonfractionated, and changed classification were assessed between segments. **b** All segments were mutually compared. In each case, an AEG was considered “stable CFAE” if the assessed segments had ICL ≥ 4; an AEG was considered “stable non-CFAE” if the assessed segments had ICL < 4; or an AEG was considered “unstable” if the AEG changed from fractionated to nonfractionated or vice versa within the assessed segments. The percentage of “stable CFAE,” “stable non-CFAE,” and “unstable AEG” in each segment was calculated. **c** The temporal decay of stable AEGs was assessed. In the first 2.5-s segment, all AEGs were considered stable. On segment 2, a total of 151 AEGs were classified as unstable. On the last segment, additional 62 AEGs changed their classification. The exponential best fit with a time constant (*τ*) of 2.8 s. **d** The regional occurrence of the different types of AEGs normalized by the number of collected points per region

The temporal behavior of the three consecutive segments for each collected point is shown in Fig. 4, considering ICL ≥ 4. Figure 4a shows that 85% of the AEGs initially classified as fractionated in segment 1 remained fractionated in segment 2, while 15% changed from fractionated to nonfractionated. Similarly, 77% of the AEGs classified as nonfractionated in segment 1 remained nonfractionated in segment 2, while 23% changed from nonfractionated to fractionated. In the following segments, 87% of AEGs classified as fractionated in segment 2 remained fractionated in segment 3, while 13% changed from fractionated to nonfractionated; 80% of the AEGs classified as nonfractionated in segment 2 remained nonfractionated in segment 3, while 20% changed from nonfractionated to fractionated.

When comparing segment 1 versus 2, 47% of the total AEGs were labeled as stable CFAEs, while 35% were stable non-CFAEs, and 18% AEGs were unstable (Fig. 4b). When comparing segment 2 versus 3, 49% of the total AEGs were labeled as stable CFAEs, 35% were stable non-CFAEs, and 16% AEGs were unstable. More importantly, 42% AEGs were stable CFAEs within the three segments, 31% were stable non-CFAEs, and 27% were unstable.

Figure 4c illustrates the temporal decay of stable AEGs. In the first 2.5-s segment, all AEGs were considered stable since it was the first classification (797 AEGs). On segment 2, a total of 151 AEGs were classified as unstable, remaining 646 stable AEGs. On the last segment, additional 62 AEGs changed their classification, remaining 584 stable AEGs. The exponential best fit suggests a temporal decay (*τ*) of 2.8 s, in which 540 AEGs (68% of 797 AEGs) would be temporally stable.

The occurrence of the different types of AEGs (stable CFAE, stable non-CFAE, and unstable AEG) per LA region, considering the three AEG segments, is shown in Fig. 4d. Stable CFAEs were observed in all regions, with the anterior wall showing the highest incidence, followed by the septum, lateral, posterior wall, roof, and PVs. Unstable AEGs were also observed in all regions, with the LPV showing the highest incidence.

Although PVI + RL reduced AEG fractionation in all regions, unstable AEGs were still present at baseline and after ablation (please see the Supplementary material). In particular, the reduction in AEG fractionation after ablation was more evident in the AEGs collected in the PVs, as well as fewer unstable AEGs were identified in these regions after PVI + RL. The exponential best fit indicated similar AEG temporal behavior before and after PVI + RL (*τ* = 2.7 and 3 s, respectively).

### Temporal consistency of AEG fractionation with different segment lengths

**Fig. 5:**
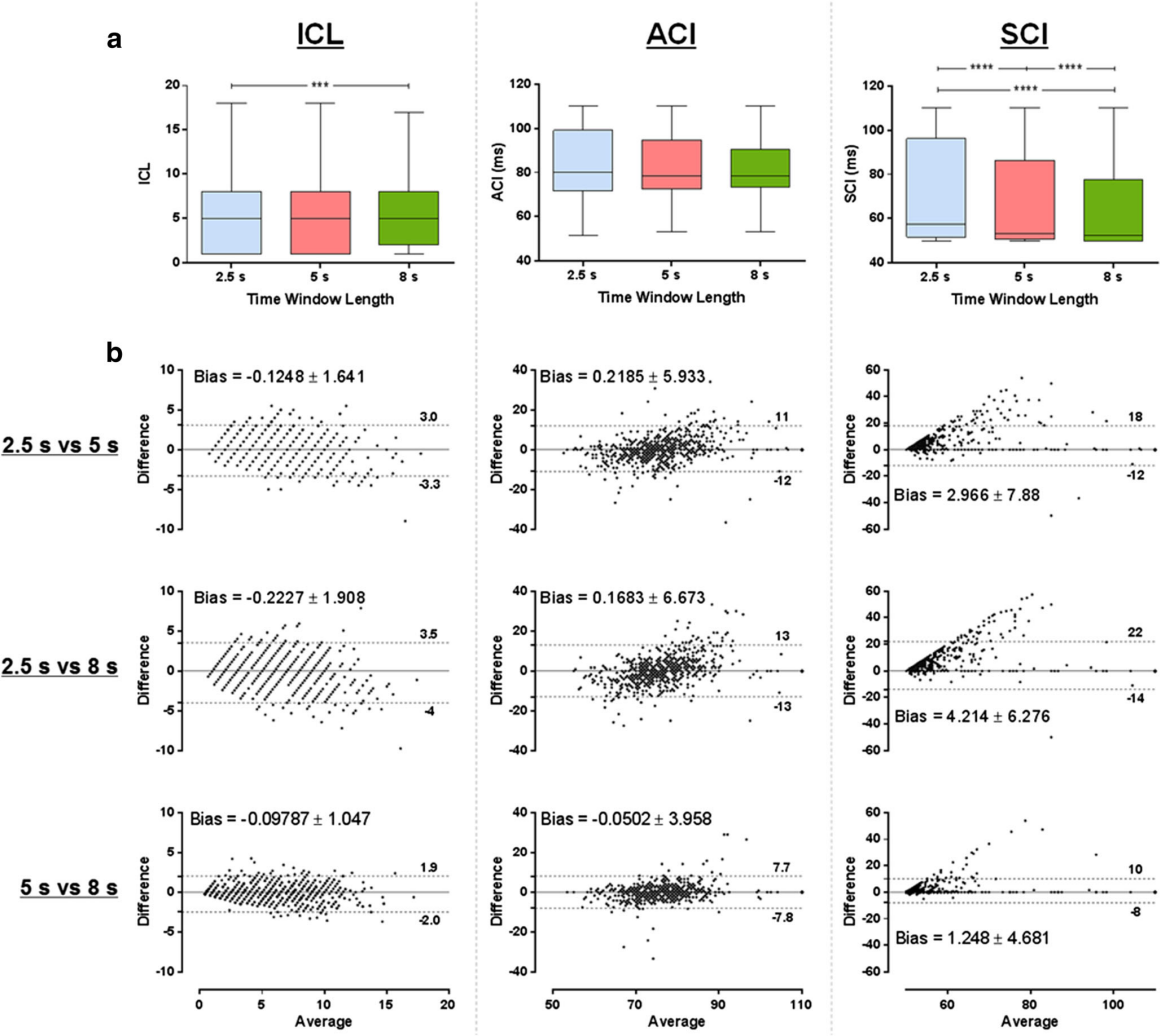
**a** The ICL, ACI, and SCI measured with 2.5, 5, and 8 s. **b** Bland-Altman plots for ICL, ACI, and SCI measured with 2.5, 5, and 8 s. ****P < 0.0001; ***P < 0.001

There was no significant difference between ICL measured with 5 versus 2.5 and 8 s, as illustrated by Fig. 5a. However, ICL measured with 2.5 s was significantly different than with 8 s. The bias calculated from the Bland-Altman plots suggests a smaller average difference between ICL calculated with 5 and 8 s when compared with the other segment lengths (2.5 vs 5 s and 2.5 vs 8 s, Fig. 5b).

Different segment lengths had little influence on ACI but significantly affected SCI (Fig. 5a). From the Bland-Altman plots, it is possible to infer that SCI calculated with longer segment lengths tends to assume smaller values. The Bland-Altman plots also suggest smaller average difference between 5 and 8 s for both ACI and SCI.

A longer AEG segment length increases the probability of a shorter complex interval to occur when compared to shorter AEG segments, which would explain the high influence of the segment lengths on the SCI. Similar results have been found when considering the data separately before and after PVI + RL (please see the Supplementary material).

**Fig. 6:**
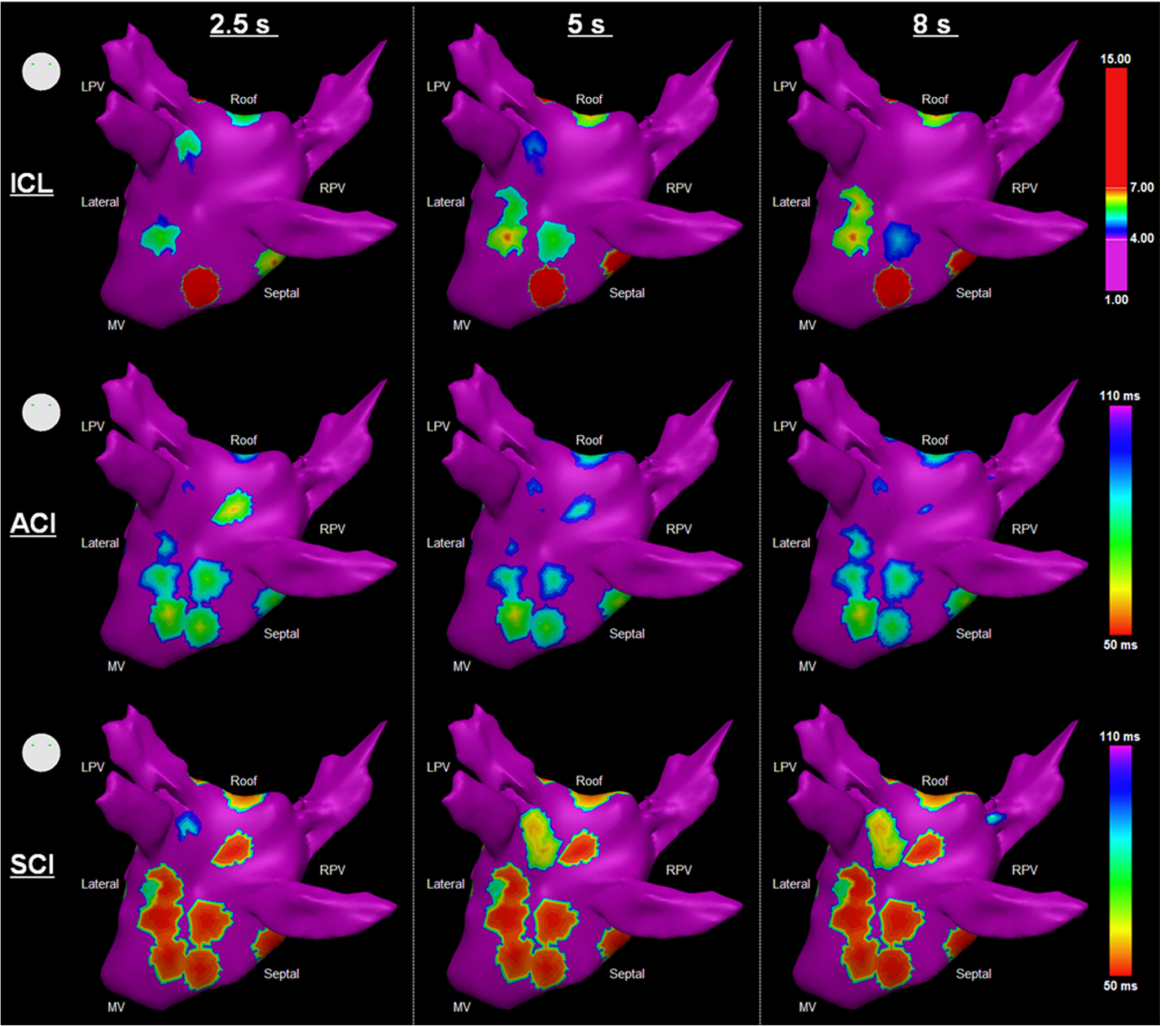
The resultant LA maps based on the different segment lengths (2.5, 5, and 8 s) for one patient as measured by ICL (*upper*), ACI (*middle*), and SCI (*bottom*)

AEG classifications were more similar between AEGs with 5- and 8-s durations, as shown in Table 3. The Spearman’s correlation was higher for 5 versus 8 s for ICL, ACI, and SCI, than 2.5 versus 5 s and 2.5 versus 8 s. Although the agreement of CFAE classification also deteriorated with higher ICL thresholds, the kappa score suggests higher agreement in the CFAE classification performed by 5- and 8-s segments in all cases. Figure 6 shows an example of the resulting CFAE map for ICL, ACI, and SCI measured with AEG durations. Maps for the remaining patients are provided in the Supplementary material.

**Table 3:** Spearman’s correlation for ICL, ACI, and SCI and kappa score for CFAE classification with different thresholds measured from the different segment lengths (2.5, 5, and 8 s)

AEG segment lengths		2.5 vs 5 s	2.5 vs 8 s	5 vs 8 s	*P* value
Spearman’s correlation (*ρ*)	ICL	0.925	0.897	0.960	<0.0001
	ACI	0.885	0.851	0.932	<0.0001
	SCI	0.872	0.818	0.921	<0.0001
Kappa score (*κ*)	ICL ≥ 4	0.836	0.780	0.874	<0.0001
	ICL ≥ 5	0.814	0.766	0.871	<0.0001
	ICL ≥ 6	0.766	0.722	0.852	<0.0001
	ICL ≥ 7	0.736	0.708	0.862	<0.0001

## Discussion

This is the first work to objectively assess the temporal behavior and consistency of AEGs using the CARTO criteria for fractionation in the same collected points. The results suggest that CARTO criteria for CFAE using 2.5-s AEGs might identify discordant atrial regions as targets for ablation depending on the moment the AEGs have been collected. Therefore, AEGs with 2.5 s are insufficient for consistent patient-specific ablation target identification, and the CARTO criteria should be revisited and consider AEG segments longer than 2.5 s for consistent CFAE classification in persAF.

### Patient-specific atrial substrate in persAF

AF sustained for long periods of time may induce structural and electrical remodeling in the atrial tissue [3]. These regions are believed to be anchored in the atrium, representing areas of remodeled atrial substrate, important in triggering and perpetuating atrial arrhythmias. AEGs acquired from such regions demonstrate low amplitude, multiple deflection activations that characterize fractionated activity due to slow or inhomogeneous conduction. The ablation of atrial regions hosting fractionated AEGs had been accepted by many as a useful additional therapy for persAF treatment [2].

With this premise, atrial regions hosting fractionated AEGs could be surrogates for atrial substrate. Therefore, targeting those regions during ablation could organize or terminate the arrhythmia [7]. However, CFAE-guided ablation has produced conflicting outcomes in previous electrophysiological studies, suggesting that not all fractionated AEG is a surrogate for atrial substrate and, therefore, not all CFAEs should be targets for ablation [17].

### The temporal behavior of AEGs during persAF

Despite much effort to understand atrial substrate properties during persAF, the dynamic nature of some AEGs continues to pose challenge for electrophysiologists in search of critical sites for ablation [8, 11, 12, 14, 15, 18].

Previous works have suggested that CFAEs demonstrate a high degree of spatial and temporal stability by analyzing consecutive CFAE maps where the AEGs for each map are collected in different time instants [8, 11, 18]. Our results, however, suggest that ablation target identification using the CARTO criteria is dependent on the time instant that the AEGs are collected; 27% of the AEGs have unstable temporal behavior, switching from fractionated to nonfractionated depending on the moment it is collected. Considering that remodeled tissue is anchored in the atria and should host “stable” fractionated activity, stable CFAE atrial sites should be considered as the premier targets for ablation, and atrial regions represented by unstable AEGs should not be targeted during ablation as they might be a result of passive wave collision from remote AF drivers and, therefore, not a true representation of atrial substrate [4, 9, 10, 16]. Ablation of those regions might create areas of slow or anisotropic conduction, thereby creating more proarrhythmogenic areas which would perpetuate the arrhythmia instead of organizing or terminating it [19]. This suggests that 27% of the collected AEGs (i.e., the unstable AEGs) could have been mistakenly ablated should STAR AF2-like guidance for CFAEs ablation be followed [17].

### The AEG duration for atrial substrate assessment

Although the NavX algorithm is based on different criteria for CFAE classification, there is no evidence that the NavX algorithm is better than CARTO’s, except perhaps for the fact that the NavX criteria allows operators to perform CFAE analysis on epochs longer than 2.5 s (and up to 8 s), which would facilitate the investigation of AEG segment lengths for measurements of CFAEs [14]. CARTO, on the other hand, fixes the AEG duration in 2.5 s, which has limited the studies on temporal consistency of AEG fractionation. Our results agree with previously published data that suggest AEG duration of 2.5 s is not sufficient to measure CFAEs consistently in persAF using the CARTO criteria [15]. However, this previous work limited the analysis to 2.5- and 5-s recording durations, while our work extends the analysis to 8 s. Additionally, we also have shown that unstable AEGs would change their state with a time constant of 2.8 s. AEGs with 5 and 8 s generated more similar CFAE maps than those created with 2.5-s AEGs. Our results show both (i) how fast the behavior based on the CARTO criteria changes—the best fit exponential shown in Fig. 4c, with a time constant of 2.8 s and (ii) how fast the results of the parameters (ICL, ACI, SCI) converge, as shown in Fig. 5b. Both results support the conclusion that AEG recording durations longer than 2.5 s should be used for CFAE classification using the CARTO criteria. Although it can be challenging to infer what is “optimal,” we would suggest that 5 s is sufficient and 7.5 s is close to the “optimum” duration for analysis based on the results shown in Fig. 5b.

### Limitations

The time consistency and short AEG epochs are not the only limitations of an otherwise accurate CARTO algorithm to identify targets for persAF ablation. On the contrary, other factors that contribute to inconsistent ablation outcomes using CARTO are as follows: the simplistic and nonphysiologic rationale behind the counting of the number of fractionated deflections, the fact that the spatial consistency (clustering) of CFAEs at neighboring locations is poorly analyzed, and the absence of analysis of other indices, such as in the frequency domain. Each factor, however, deserves dedicated investigation and is out of the scope of the present work.

We acknowledge that some of the patients in the study were taking amiodarone, which could potentially affect AEG fractionation. Nevertheless, our results would still be applicable considering amiodarone is frequently administered to persAF patients undergoing ablation.

We also acknowledge that, from the nature of the proposed study design, it is challenging to infer about the optimal AEG duration for CFAE classification. In the present study, the maximum AEG duration was limited to 8 s by the existing devices. Naturally, longer AEG recording durations would facilitate the investigation of the “optimum” segment length for proper CFAE classification. Unfortunately, few—if none—devices permit such analysis, and the results found in the present work are relevant and timely, as they can be applied with the technology currently available.

The preset study involved a small number of patients, and additional points would help to validate the results. However, as the main objective of this study was to investigate the spatiotemporal behavior of AEGs according the CARTO criteria during persAF, we do believe that this limitation is partially overcome considering the number of points collected from the 18 patients, providing information from a balanced distribution of different LA anatomical sites, as illustrated in Fig. 4d. Further studies with a more representative population need to be performed to consolidate which measurement is better or can get better ablation outcome, as well as to investigate whether CFAE maps with longer recording durations correspond to fibrosis area estimated from image analysis, such as late gadolinium enhancement of LA in magnetic resonance imaging.

## Conclusions

This study investigated the temporal behavior of AEGs collected during persAF and the temporal consistency of AEG fractionation considering different AEG segment lengths using the CARTO criteria for fractionation in the same collected points. Our findings demonstrate that the CARTO CFAE criteria has been used in the clinical practice in the last 10 years considering insufficient recording duration to detect CFAEs. The resulting CFAE maps created with CARTO using 2.5-s AEG epochs are dependent on the time instant that the AEGs have been collected. Therefore, ablation will target different atrial regions depending on the moment that the CFAE maps have been created, which contributes to the conflicting outcomes in persAF ablation reported in previous works. Our results show that CARTO criteria should be revisited and consider longer recording segments for consistent CFAE classification.

## Electronic supplementary material

ESM 1 (DOCX 11813 kb)
