# Supplementary material for: The temporal behavior and consistency of bipolar atrial electrograms in human persistent atrial fibrillation
Source: Med Biol Eng Comput. 2017 Jul 3;56(1):71–83. doi: 10.1007/s11517-017-1667-1 (PMC5766733; doi:10.1007/s11517-017-1667-1)
Supplement: Supplementary file 1 — (DOCX 11813 kb) [file 11517_2017_1667_MOESM1_ESM.docx]

**The temporal behaviour and consistency of bipolar atrial electrograms in**

**human persistent atrial fibrillation**

**Supplemental Material***

***intended for publication as an online data supplement**

Tiago P Almeida, PhD*; Gavin S Chu, MB BChir, MA(Cantab), MRCP(UK)‡§; Michael J Bell, BEng*; Xin Li, MSc*; João L Salinet, PhD†; Nawshin Dastagir, BEng*; Jiun H Tuan, MB BS, MD, MRCP§; Peter J Stafford, MB BS, MD, FRCP§; Fernando S Schlindwein, PhD, DSc*#; G André Ng, MBChB, PhD, FRCP(Glasg), FRCP, FESC‡§#

*Department of Engineering, University of Leicester, UK; ‡Department of Cardiovascular Science, University of Leicester, UK; §University Hospitals of Leicester NHS Trust, UK; †Biomedical Engineering, Engineering, Modelling and Applied Social Sciences Centre, Federal ABC University, Brazil; #National Institute for Health Research Leicester Cardiovascular Biomedical Research Centre, Glenfield Hospital, UK.

**Address for correspondence:**

Dr. Fernando S. Schlindwein,

Department of Engineering, University of Leicester,

University Road, Leicester, LE1 7RH, England, UK

Email: f.s.schlindwein@leicester.ac.uk

Tel: +44 (0)116 252 5053

Fax: +44 (0)116 252 2619

**Methods**

***Study population***

**Table S1.** Clinical characteristics of study population (N = 18).

| Age, yrs |  | 56.1 ± 9.3 |
| --- | --- | --- |
| Male/Female |  | 16/2 |
| History of AF, months |  | 67.2 ± 45.6 |
| Ejection Fraction,% |  | 48 ± 1 |
| Left Atrial Diameter, mm |  | 47 ± 1 |
| History of coronary artery disease |  | 4 |
| Medication* (number of patients on) |  |  |
| ACE inhibitor / ARB |  | 11 |
| Amiodarone |  | 10 |
| Beta-blockers |  | 8 |
| Calcium channel blockers |  | 2 |
| Digoxin |  | 1 |
| Sotalol |  | 5 |

Values are mean ± SD or n.

* All anti-arrhythmic and rate-controlling drugs were stopped for at least five half-lives before the procedure, with the exception of amiodarone. ACE = angiotensin-converting-enzyme; AF = atrial fibrillation; ARB = angiotensin receptor blockers.

**Results**

***Temporal behavior of consecutive AEGs before PVI + RL***

The temporal behavior of the three consecutive segments for each collected point prior PVI + RL is shown on Figure S1. Figure S1A shows that 86% of the AEGs initially classified as fractionated in segment 1 remained fractionated in segment 2, while 14% changed from fractionated to non-fractionated. Similarly, 67% of the AEGs classified as non-fractionated in segment 1 remained non-fractionated in segment 2, while 33% changed from non-fractionated to fractionated. In the following segments, 88% of AEGs classified as fractionated in segment 2 remained fractionated in segment 3, while 12% changed from fractionated to non-fractionated. 74% of the AEGs classified as non-fractionated in segment 2 remained non-fractionated in segment 3, while 26% changed from non-fractionated to fractionated. When comparing segment 1 versus 2, 57% of the total AEGs before any ablation were labelled as stable CFAEs, while 23% were stable non-CFAEs, and 20% AEGs were unstable (Figure S1B). When comparing segment 2 versus 3, 60% of the total AEGs were labelled as stable CFAEs, 24% were stable non-CFAEs, and 16% AEGs were unstable. A total of 52% AEGs were stable CFAEs within the three segments, 20% were stable non-CFAEs and 26% were unstable.

Figure S1C illustrates the temporal decay of stable AEGs (CFAEs and non-CAFE) before PVI + RL. In the first 2.5 s segment, all AEGs were considered stable since it was the first classification (455 AEGs). On segment 2, a total of 92 AEGs were classified as unstable, remaining 363 stable AEGs. On the last segment, additional 36 AEGs changed their classification, remaining 327 stable AEGs. The exponential best fit suggests a temporal decay (τ) of 2.7 s, in which 304 AEGs (67% of 455 AEGs) would be temporally stable prior PVI + RL.


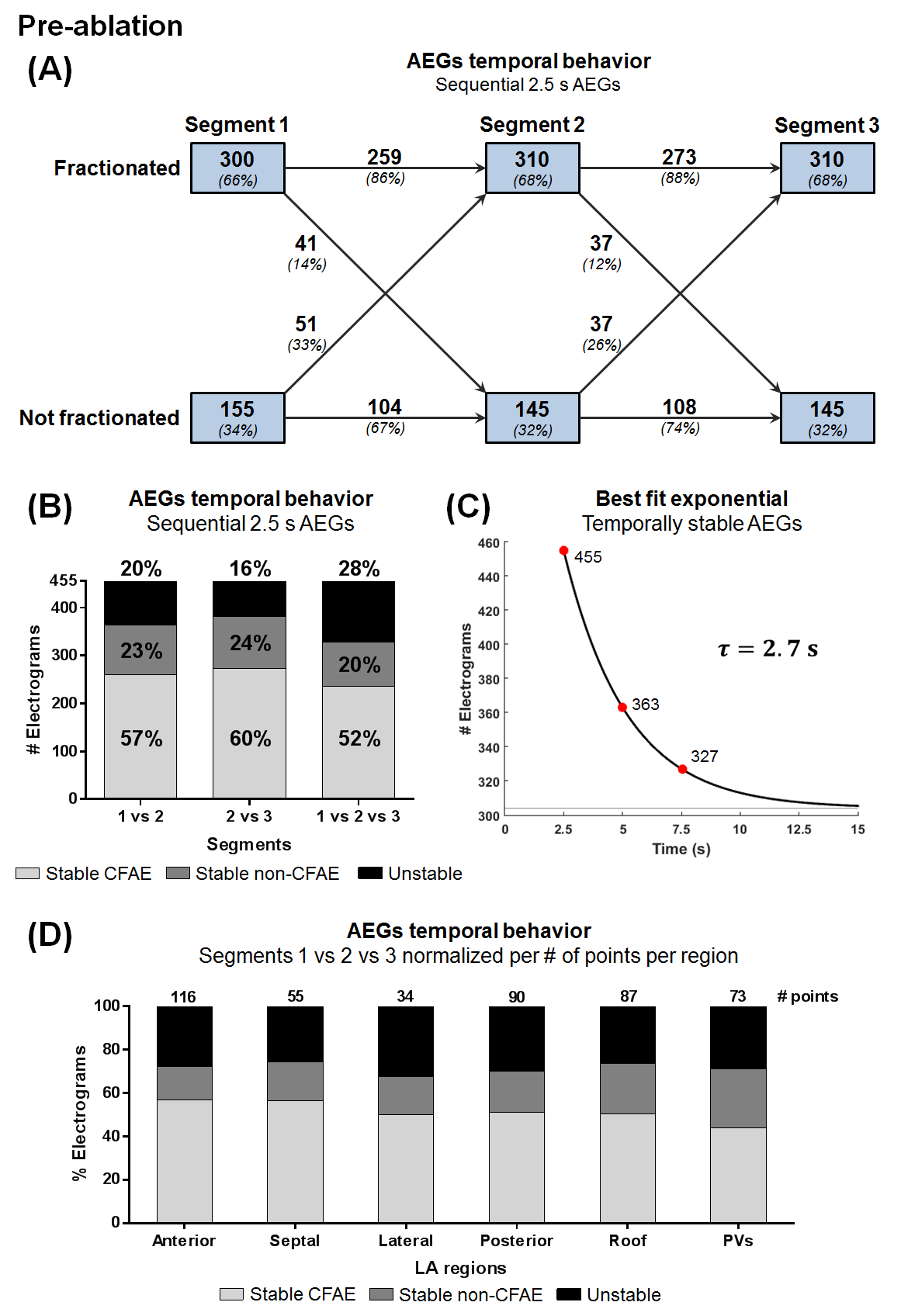


**Figure S1.** The temporal behavior of three consecutive AEG segments with 2.5 s duration each prior PVI + RL. A. The 8 s segments were divided in three consecutive 2.5 s length segments. The ICL was measured in each segment and classified whether as fractionated or non-fractionated. The AEGs that remained fractionated, remained non-fractionated and changed classification were assessed between segments. B. The percentage of ‘stable CFAE’; ‘stable non CFAE’ and ‘unstable AEG’ calculated in each segment. C. The temporal decay of stable AEGs assessed within 7.5 s. D. The regional occurrence of the different types of AEGs normalized by the number of collected points per region.

The occurrence of the different types of AEGs (stable CFAE, stable non-CFAE and unstable AEG) per LA region before ablation, considering the three AEG segments, is shown in Figure S1D. Stable CFAEs were observed in all regions, with the anterior wall showing the highest incidence, followed by the septum, posterior wall, lateral, roof and PVs. Unstable AEGs were also observed in all regions, with the lateral showing the highest incidence.

***Temporal behavior of consecutive AEGs after PVI + RL***

The temporal behavior of the three consecutive segments for each collected point after PVI + RL is shown on Figure S2. Figure S2A shows that 81% of the AEGs initially classified as fractionated in segment 1 remained fractionated in segment 2, while 19% changed from fractionated to non-fractionated. Similarly, 84% of the AEGs classified as non-fractionated in segment 1 remained non-fractionated in segment 2, while 16% changed from non-fractionated to fractionated. In the following segments, 83% of AEGs classified as fractionated in segment 2 remained fractionated in segment 3, while 17% changed from fractionated to non-fractionated. 87% of the AEGs classified as non-fractionated in segment 2 remained non-fractionated in segment 3, while 13% changed from non-fractionated to fractionated. When comparing segment 1 versus 2, 32% of the total AEGs after ablation were labelled as stable CFAEs, while 51% were stable non-CFAEs, and 17% AEGs were unstable (Figure S2B). When comparing segment 2 versus 3, 35% of the total AEGs were labelled as stable CFAEs, 50% were stable non-CFAEs, and 15% AEGs were unstable. A total of 28% AEGs were stable CFAEs within the three segments, 47% were stable non-CFAEs and 25% were unstable.

Figure S2C illustrates the temporal decay of stable AEGs (CFAEs and non-CAFE) after PVI + RL. In the first 2.5 s segment, all AEGs were considered stable since it was the first classification (342 AEGs). On segment 2, a total of 59 AEGs were classified as unstable, remaining 283 stable AEGs. On the last segment, additional 26 AEGs changed their classification, remaining 257 stable AEGs. The exponential best fit suggests a temporal decay (τ) of 3 s, in which 236 AEGs (69% of 342 AEGs) would be temporally stable after PVI + RL.


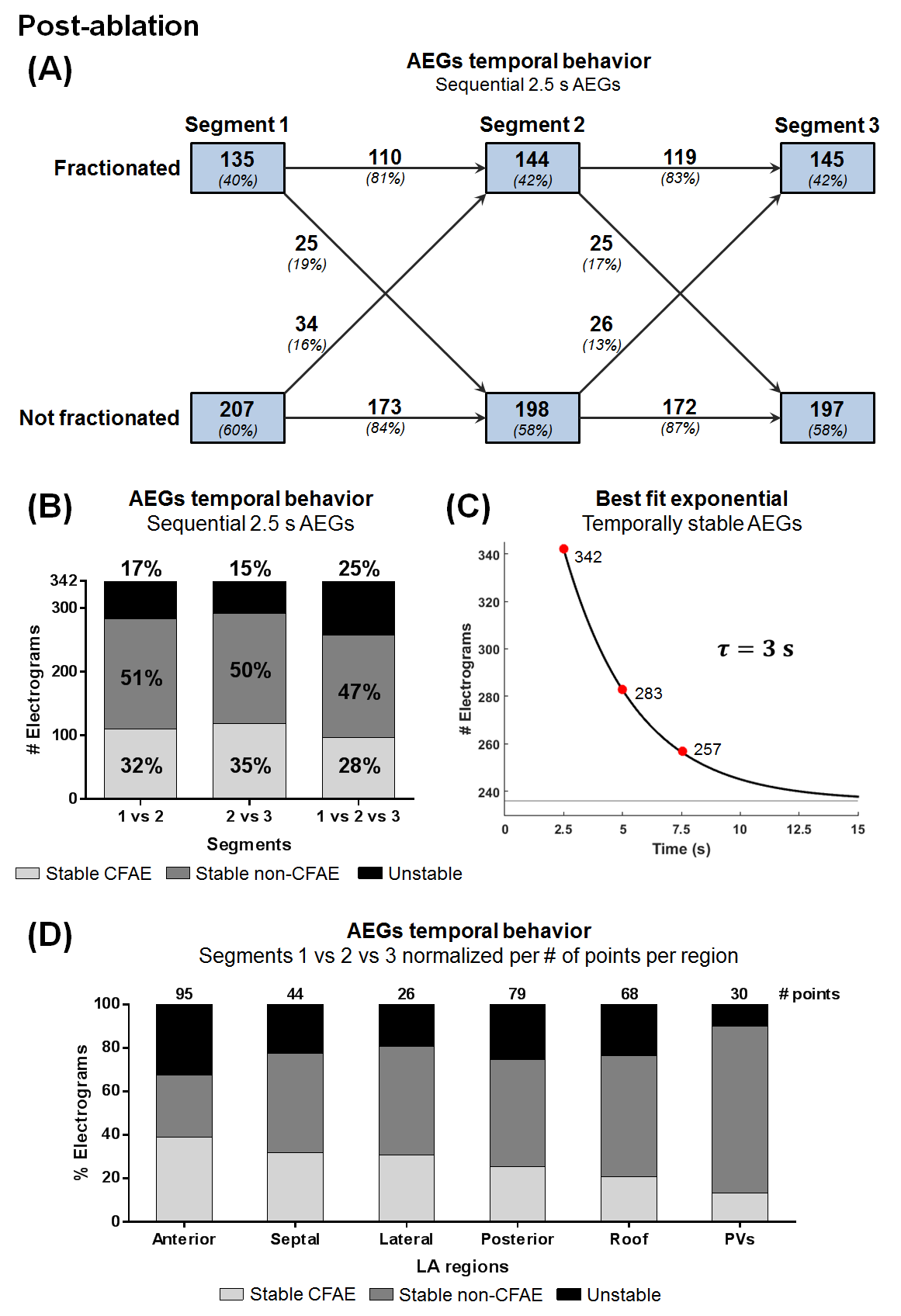


**Figure S2.** The temporal behavior of three consecutive AEG segments with 2.5 s duration each after PVI + RL. A. The 8 s segments were divided in three consecutive 2.5 s length segments. The ICL was measured in each segment and classified whether as fractionated or non-fractionated. The AEGs that remained fractionated, remained non-fractionated and changed classification were assessed between segments. B. The percentage of ‘stable CFAE’; ‘stable non CFAE’ and ‘unstable AEG’ calculated in each segment. C. The temporal decay of stable AEGs assessed within 7.5 s. D. The regional occurrence of the different types of AEGs normalized by the number of collected points per region.

The occurrence of the different types of AEGs (stable CFAE, stable non-CFAE and unstable AEG) per LA region after ablation, considering the three AEG segments, is shown in Figure S2D. Stable CFAEs were observed in all regions, with the anterior wall showing the highest incidence, followed by the septum, posterior wall, lateral, roof and PVs. Unstable AEGs were also observed in all regions, with the anterior wall showing the highest incidence.

***Temporal consistency of AEG fractionation with different segment lengths before PVI + RL***

ICL measured with 2.5 s 5 s and 8 s before ablation is illustrated in Figure S3A. ICL measured with 2.5 s was significantly different than with 5 s and 8 s. The bias calculated from the Bland-Altman plots suggests a smaller average difference between ICL calculated with 5 s and 8 s when compared with the other segment lengths (2.5 s vs 5 s and 2.5 s vs 8 s, Figure S3B).

Different segment lengths had little influence on ACI prior ablation, but significantly affected SCI (Figure S3A). Nevertheless, the Bland-Altman plots also suggest smaller average difference between 5 s and 8 s for both ACI and SCI (Figure S3B).

***Temporal consistency of AEG fractionation with different segment lengths after PVI + RL***

Different segment lengths had little influence on ICL measured with 2.5 s 5 s and 8 s after ablation is illustrated in Figure S4A. The bias calculated from the Bland-Altman plots suggests a smaller average difference between ICL calculated with 5 s and 8 s when compared with the other segment lengths (2.5 s vs 5 s and 2.5 s vs 8 s, Figure S4B).

Different segment lengths also had little influence on ACI after ablation, but significantly affected SCI (Figure S4A). Nevertheless, the Bland-Altman plots also suggest smaller average difference between 5 s and 8 s for both ACI and SCI (Figure S4B).


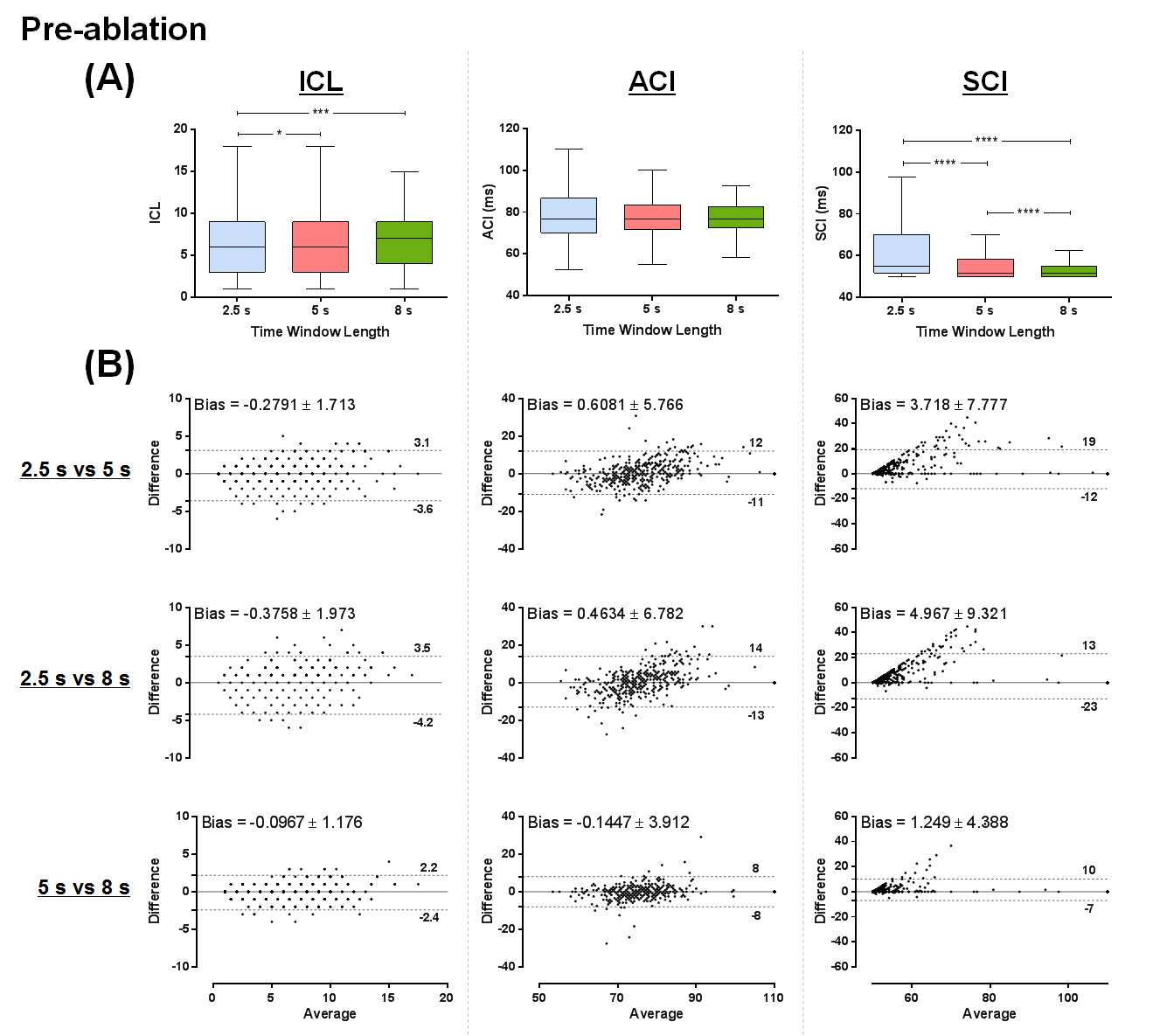


**Figure S3.** The ICL, ACI and SCI measured with 2.5 s, 5 s and 8 s prior ablation. B. Bland-Altman plots for ICL, ACI and SCI measured with 2.5 s, 5 s and 8-s. **** P<0.0001; *** P<0.001; * P<0.05.


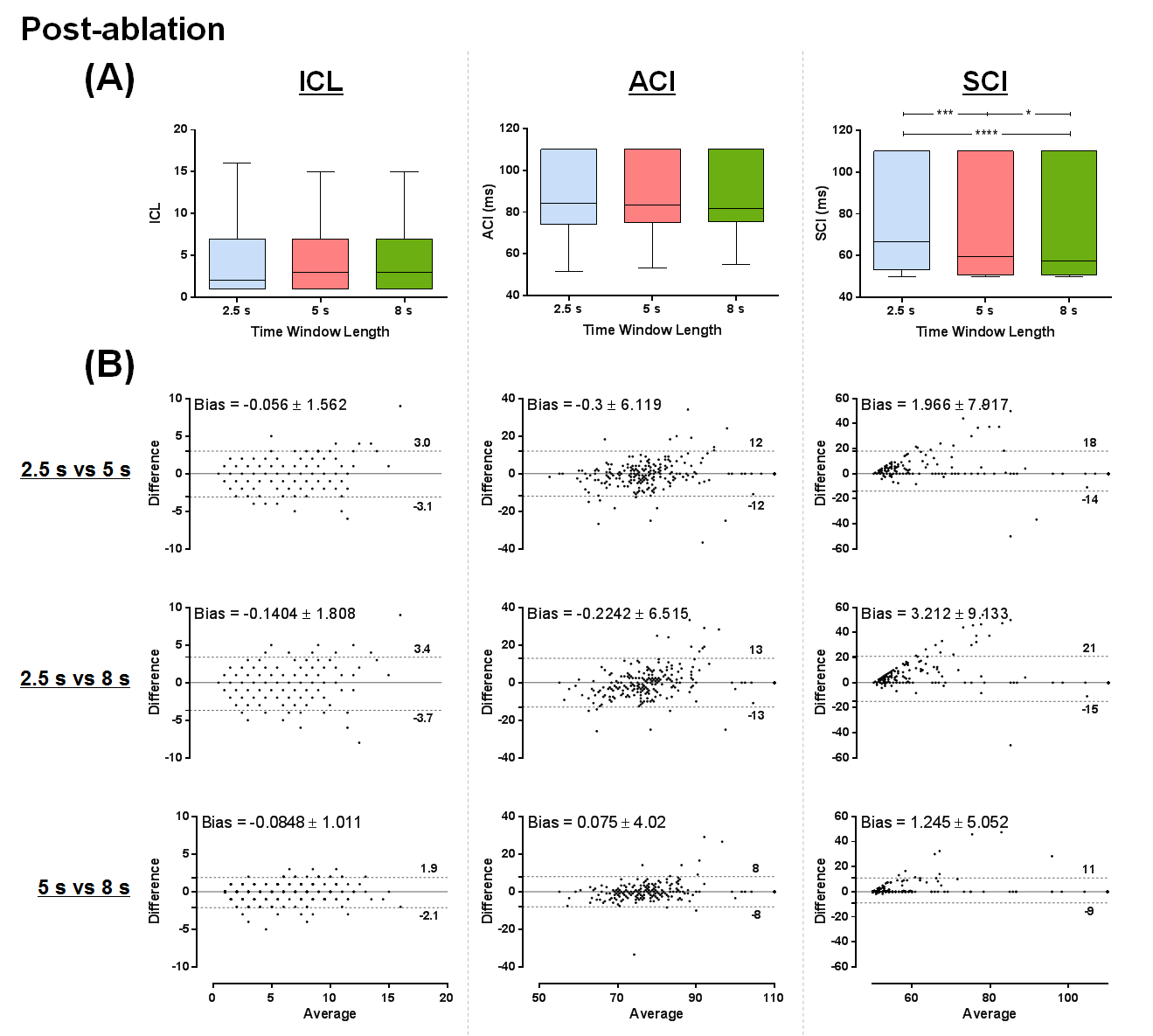


**Figure S4.** The ICL, ACI and SCI measured with 2.5 s, 5 s and 8 s after ablation. B. Bland-Altman plots for ICL, ACI and SCI measured with 2.5 s, 5 s and 8-s. **** P<0.0001; *** P<0.001; * P<0.05.

The LA maps based on the three consecutive AEG segments with 2.5 s duration each for all patients as measured by ICL, ACI and SCI are shown on Figures S5, S6 and S7, respectively.


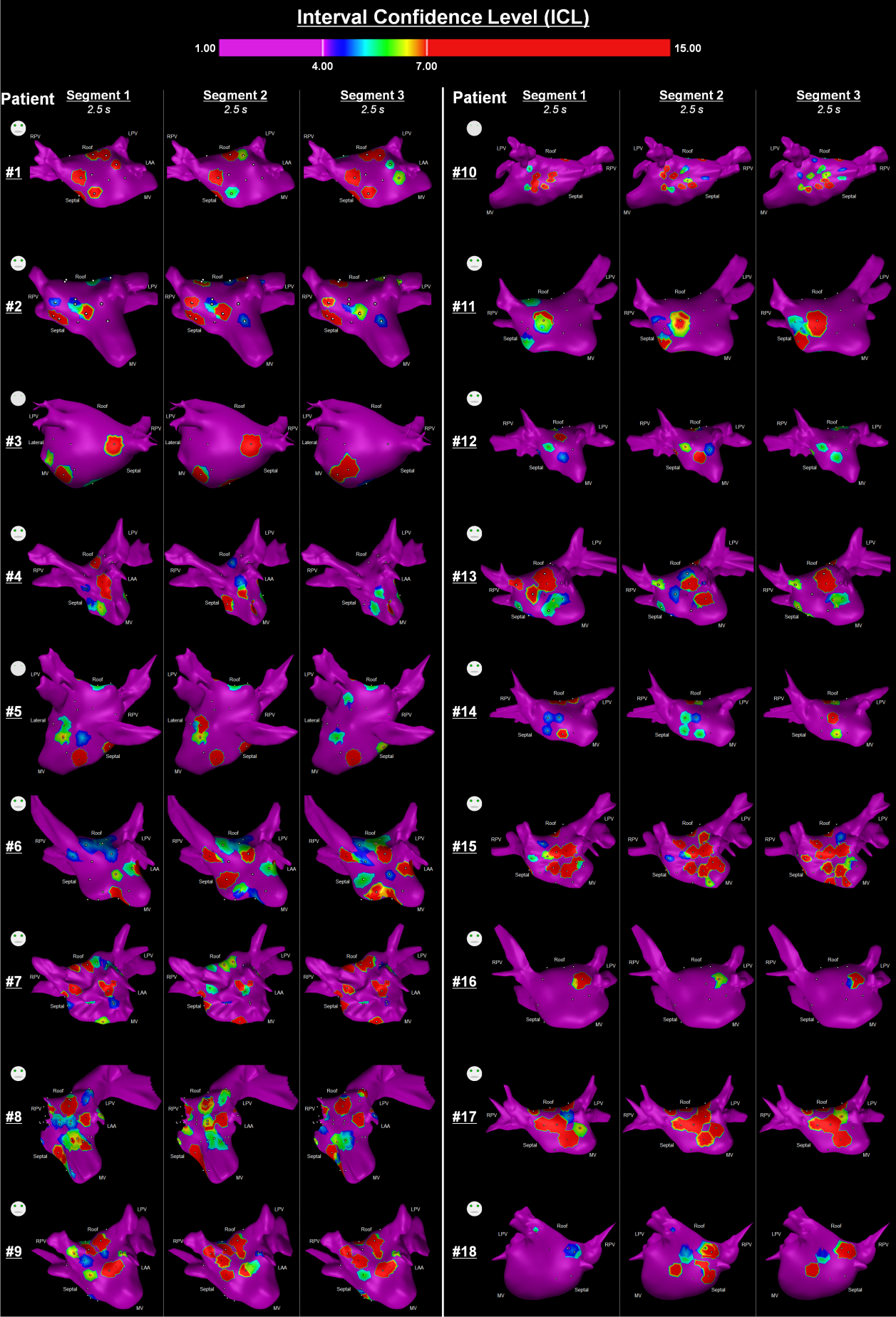


**Figure S5.** The three consecutive AEG segments with 2.5 s duration measured by ICL.


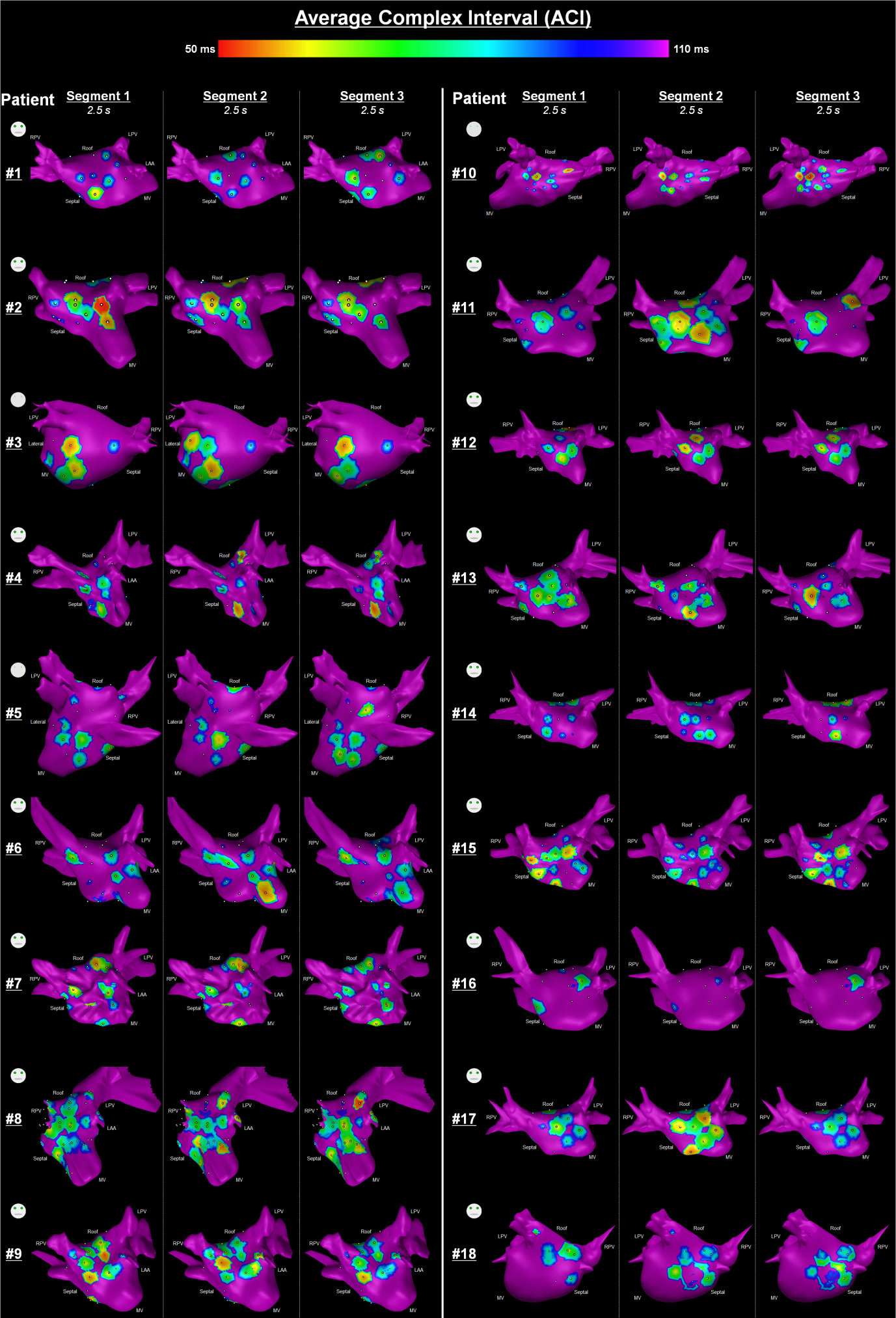


**Figure S6.** The three consecutive AEG segments with 2.5 s duration measured by ACI.


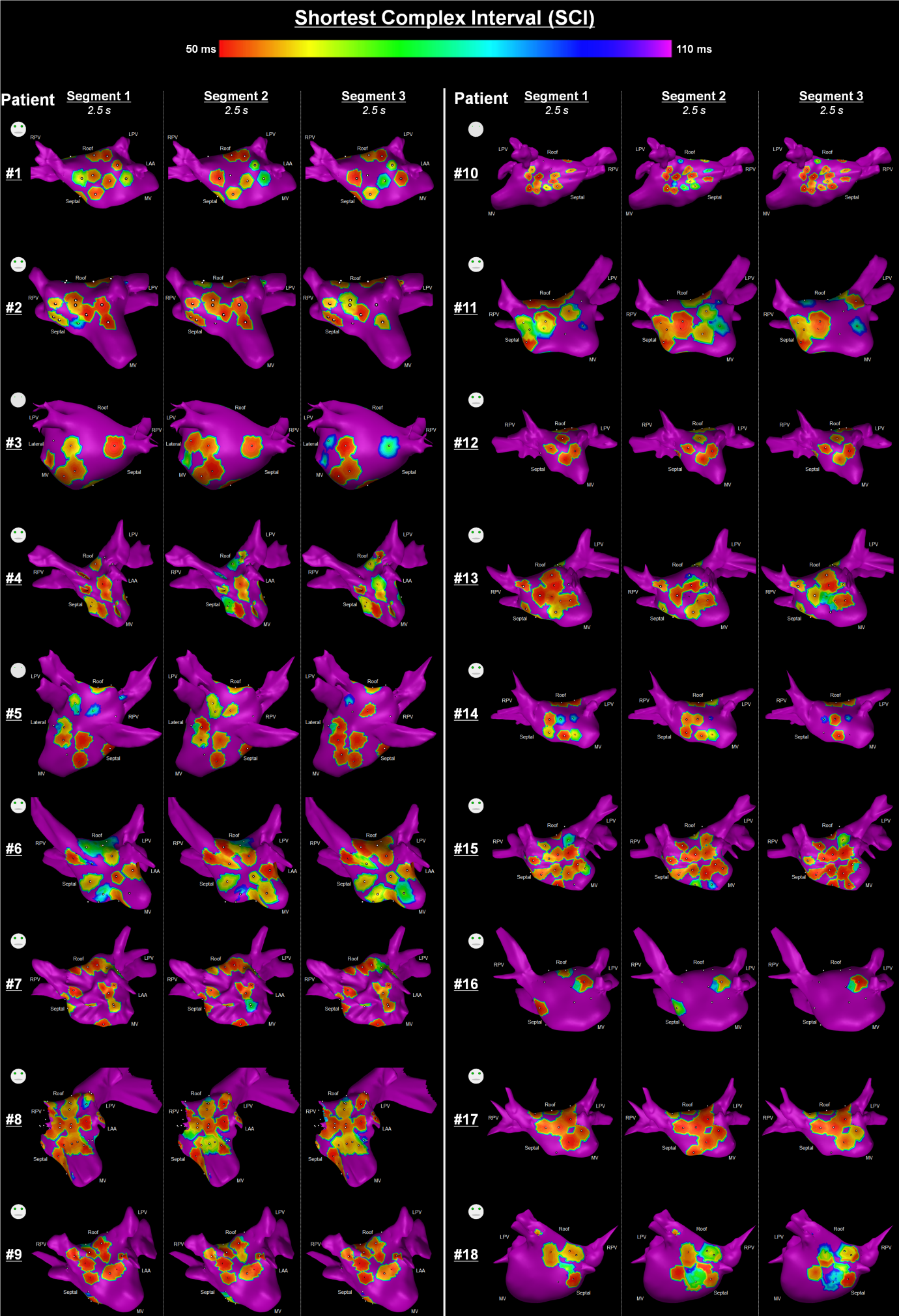


**Figure S7.** The three consecutive AEG segments with 2.5 s duration measured by SCI.

The LA maps based on the on the different segment lengths (2.5 s, 5 s and 8 s) for all patients as measured by ICL, ACI and SCI are shown on Figures S8, S9 and S10, respectively.


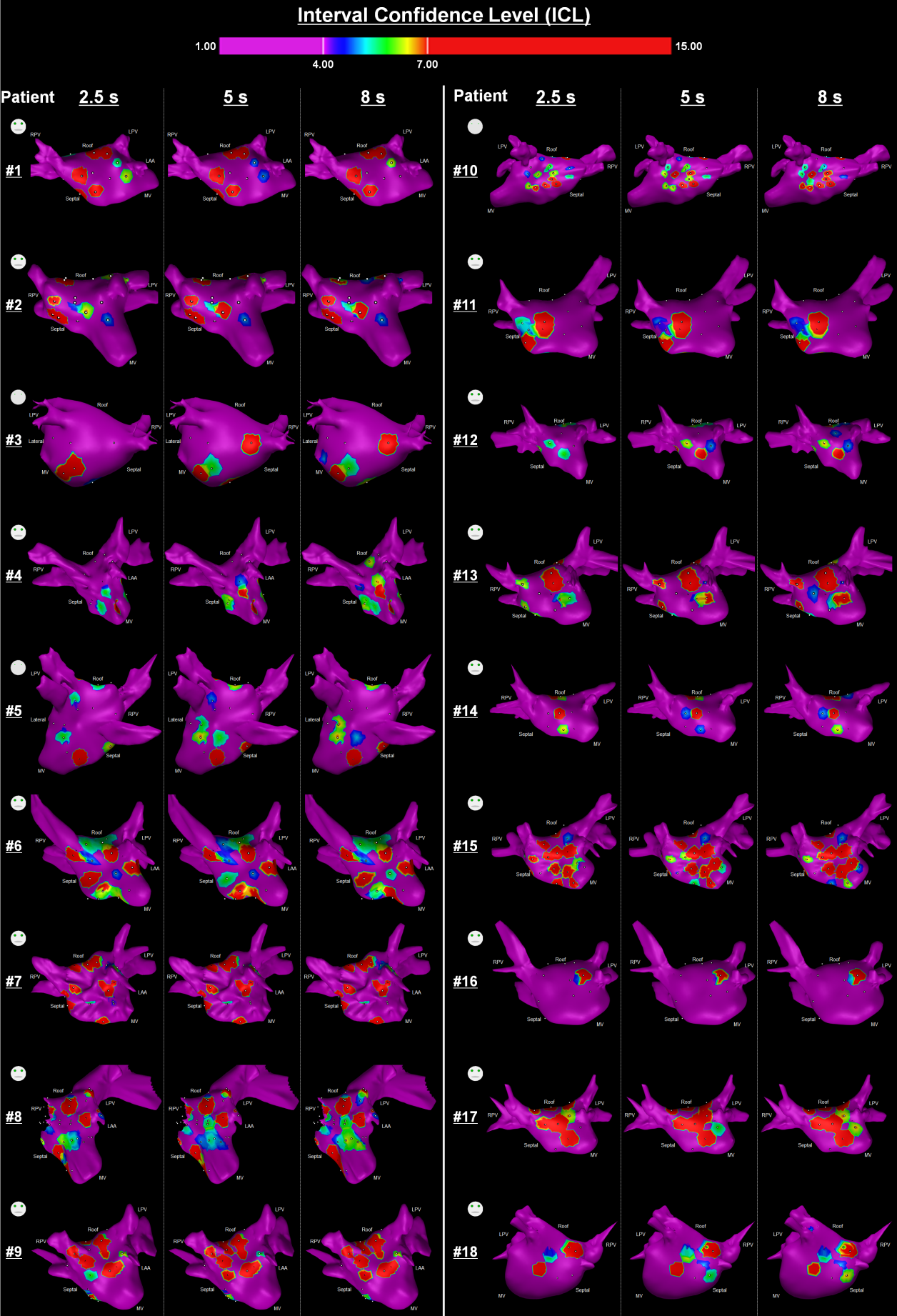


**Figure S8.** The different segment lengths (2.5 s, 5 s and 8 s) measured by ICL.


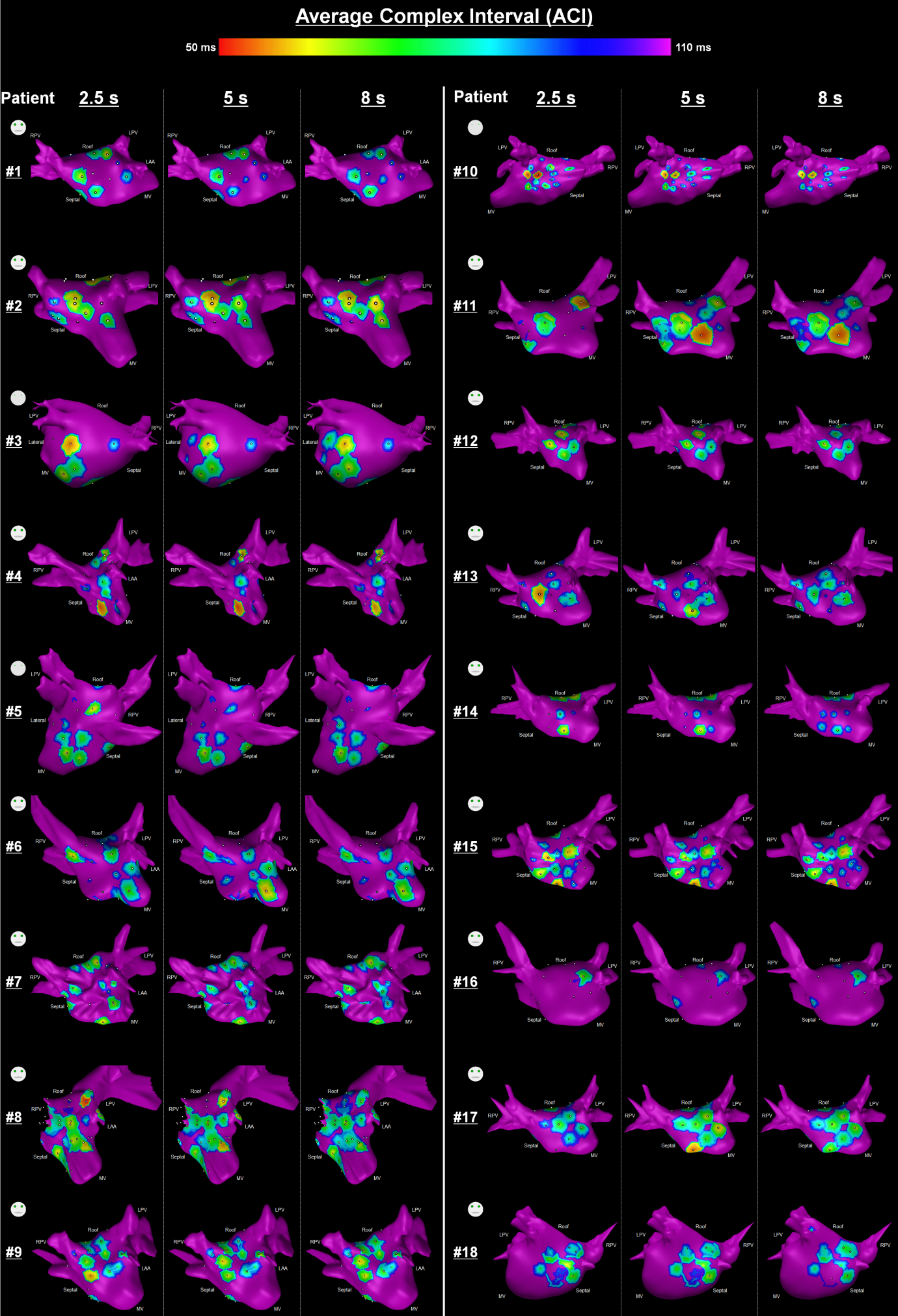


**Figure S9.** The different segment lengths (2.5 s, 5 s and 8 s) measured by ACI.


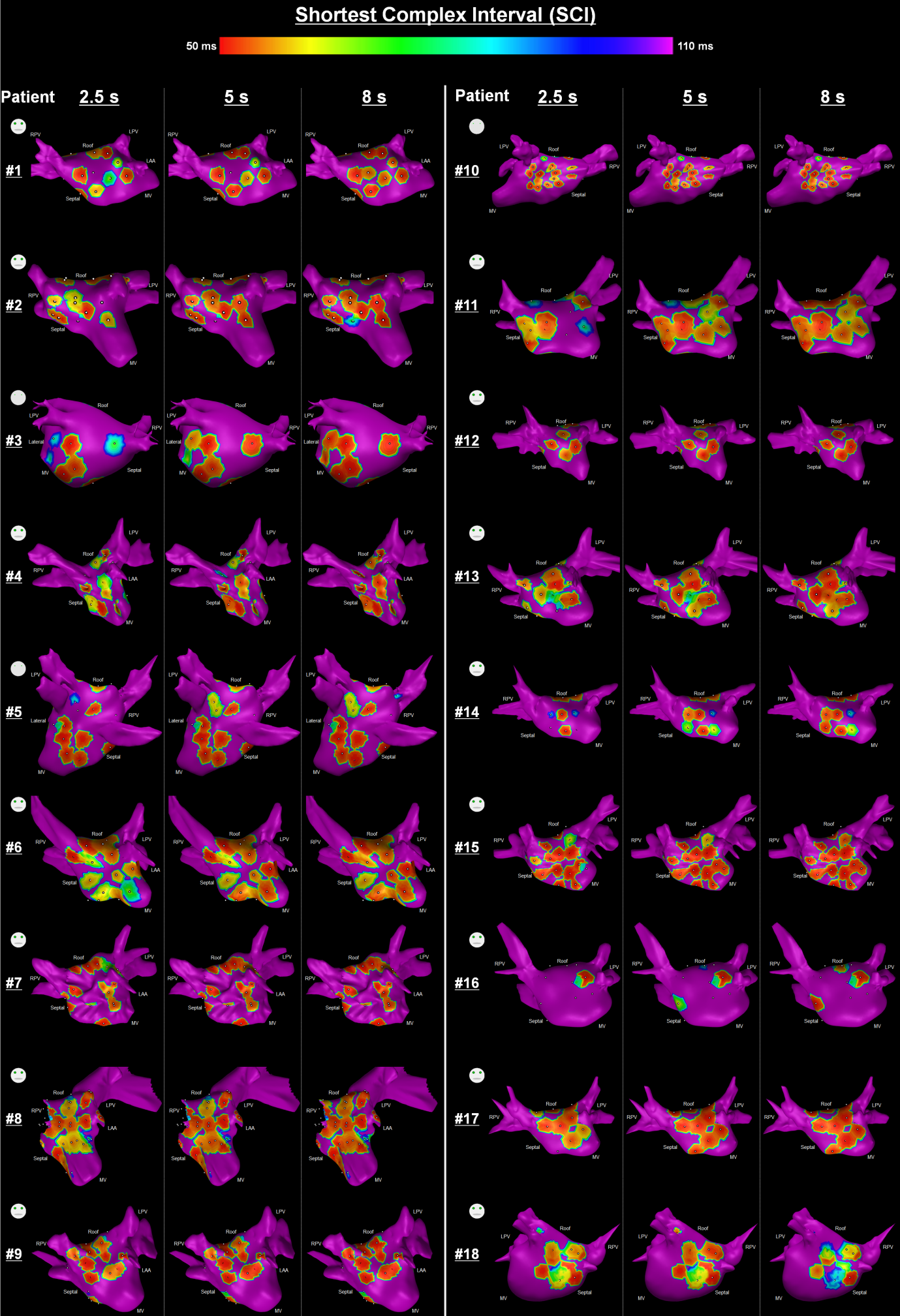


**Figure S10.** The different segment lengths (2.5 s, 5 s and 8 s) measured by SCI.
